# The Impact of Heat on Health and Productivity among Maize Farmers in a Tropical Climate Area

**DOI:** 10.1155/2019/9896410

**Published:** 2019-04-01

**Authors:** Lukman Shiji Sadiq, Zailina Hashim, Malina Osman

**Affiliations:** ^1^Department of Environmental and Occupational Health, Faculty of Medicine and Health Sciences, 43400 Serdang, Malaysia; ^2^Department of Microbiology and Parasitology, Faculty of Medicine and Health Sciences, UPM, Seri Kembangan, Malaysia

## Abstract

**Background:**

Heat stress disorders may cause negative health outcome and subsequent productivity reduction especially in those who work under direct sunlight for an extended number of hours.

**Objective:**

This study assessed the impact of heat on the health and productivity among maize farmers in a hot tropical country.

**Methods:**

A cross-sectional study was conducted among 396 maize farmers, randomly selected across Gombe province, Nigeria. The wet bulb globe temperature monitor (WBGT) Model QuesTemp^0^36 was used in determining the heat index. Health was determined using a validated questionnaire, while productivity was determined by recording work output based on the number of ridges cultivated during the working hours.

**Results:**

The farms recorded mean heat index with standard deviation (SD) of 31.56 (2.19) and 34.08 (1.54) in the hours of 9 am to 12 pm and 12–3 pm respectively, which exceeded the threshold level set by the ACGIH. Heavy sweating (93.2%), tiredness (48.5%), dizziness (34.1%), and headache (40.4%) were experienced by the respondents almost on daily basis. The finding further showed a significant difference in the farmers' productivity during the three time duration of the work day (*p* < 0.001). The productivity was significantly higher between the hours of 6–9 am (*p* < 0.001) and 12–3 pm (*p* < 0.001), compared to the hours of 9 am to 12 pm (*p* < 0.001). The factors that significantly predict the productivity outcome include temperature (*p* < 0.001), gender (*p* < 0.001), age (*p*=0.033), and BMI (*p*=0.008).

**Conclusion:**

The farmers were frequently experiencing heat exhaustion which decreased their productivity.

## 1. Introduction

Climate change is one of the greatest threats facing the globe because it causes a rise in the ambient temperature of our environment [1] and also a major threat to sensitive sectors such as agriculture and animal husbandry [2]. The Intergovernmental Panel on Climate Change (IPCC) confirms that the global average surface temperature shows a warming of about 0.85°C over the period of 1880 to 2012 [3]. Additionally, with the continued emission of greenhouse gases at the year 2000 levels, a further warming of about 1°C per decade would be expected. Furthermore, currently, each of the first six months of 2016 set a record as the warmest respective month globally in the modern temperature record, which dates to 1880 [4]. Exposure to heat when WBGT exceeds 26–30°C can reduce work capacity and cause serious health problems such as sun stroke, muscle cramps, heat exhaustion, heat stroke, and even death, depending on the humidity, wind movement, and heat radiation. People within the group of low- and middle-income countries are more vulnerable because many of them engage in heavy physical work, either outdoors under direct sunlight or indoors without effective cooling [5]. Farming operations carried out by farm workers are normally at high risk of heat stress, as they work under high pressure, perform extended hours of work under direct sunlight and high temperature, suffer dehydration, and often do not have sufficient knowledge regarding prevention from heat exposure [6]. Farmers have been among the outdoor occupational workers facing high physical load and are most at risk of severe heat exposure [7]. This kind of study such as in Nigeria, a developing country, is in limited number compared to developed nations [8]. The entire northeastern Nigeria is characterized by a high temperature that may cause heat stress due to hot and humid weather starting from May to October [9]. Gombe is among the region experiencing high temperature with about 30°C as annual mean temperature and 36–40°C maximum temperature [10]. Farmers in Gombe spent almost 8 hours per day under direct sunlight in the farming operations, and no study of this nature in particular was conducted in Gombe. This study obtained data on the health and productivity of maize farmers in Gombe, Nigeria. The study aims to determine the reported heat stress-related illnesses and symptoms and the predictors that determine the productivity of the farmers.

## 2. Materials and Methods

### 2.1. Study Area and Design

The study was conducted in Gombe State, northeastern part of Nigeria which lies within the coordinates of latitude 09°30′ and 12°30′N and longitude 08°05′ and 11°045′E. The study adopted a cross-sectional design where 396 maize farmers between 15 and 60 years of age that have farmed for two years in Gombe State, Nigeria, were followed and observed for a period of two months (July to September, 2016). The respondents were homogeneous based on their work schedule, nature, and type of activity. The sampling method used for this study was simple random sampling where respondents were randomly selected. Stat trek random generation table was used in assigning random numbers to the respondents based on the list of maize farmers obtained from the Gombe State Agricultural Development Board that serves as the sample frame. The sample size for this study was determined using the formula for calculating proportions based on proportions from previous studies [11].

(1) $$\begin{array}{c} n=\frac{Z_{1-\left(\alpha /2\right)}\sqrt{\left(2P\left(1-P\right)\right)}+Z_{1-\beta }\sqrt{{\left(P_{1}\left(1-P_{1}\right)+P_{2}\left(1-P_{2}\right)\right)}^{2}}}{{\left(P_{1}-P_{2}\right)}^{2}}, \end{array}$$

where *n* = estimated sample size, *Z*_1−(*α*/2)_=*Z* statistics for the confidence of 95% = 1.96, *Z*_1−*β*_=*Z* statistics for the power of 80% = 0.842, *P*_1_ = proportion of high knowledge level on heat stress among male = 57.0% [12], *P*_2_ = Proportion of high knowledge level on heat stress among female = 42.3% [12], *P*=((*P*_1_+*P*_2_)/2)=0.570+0.423=(0.993/2)=0.4965, and $n=\left(\left(\left(1.96\sqrt{2\;*\;0.4965\left(1-0.5\right)}\right)+\left(0.842\sqrt{0.570\left(1-0.570\right)}\right)+\left(0.423{\left(1-0.423\right)}^{2}\right)\right)/{\left(0.570-0.423\right)}^{2}\right)$.

Maximum sample size per group was *n*=180. For estimated nonrespondents, 10% of this total gives 18 added to 180, which will give a total of 198. Multiplying by design effect of 2 will give **396**.

### 2.2. Survey Instrument

A five-point Likert questionnaire for reported heat stress-related illnesses and symptoms ranged from 1 (never) to 5 (very often) was used for the data collection [13]. The questionnaire was designed based on cross reference with previous studies conducted by Thi et al. [14] and in accordance with HOTHAPS (High Occupational Temperature Health and Productivity Suppression) [15, 16]. This is to ensure that the items are in line with the objectives of the study and easily understood by the respondents.

The first section of the questionnaire collected information on sociodemographic characteristics and anthropometric data of the respondents which include gender, age, weight, and height. The second section consists of data on health symptoms of heat stress ranging from heavy sweating and tiredness/weakness to severe symptoms such as difficulty in breathing and unconsciousness. The questionnaire was validated by a panel of experts based on recommendation by Creswell [17], which comprises researchers from a local university in Malaysia. The experts have removed and added other items to the construct of the adapted questionnaire, used for determining self-reported heat stress-related symptoms, making it thirteen items instead of the initial ten. The construct validity was determined using interitems correlation between the items where the correlation coefficient (*r*) between each of them is greater than 0.3. Furthermore, the construct validation was also examined using exploratory factor analysis where the factor loading of each item is above 0.3. This shows that the items measuring the construct of experience on heat stress-related symptoms and illnesses were valid. A pilot study was conducted to test the reliability of the questionnaire and to ensure that its wordings are understood by the respondents. The Cronbach's alpha value of the pilot study and the actual study are 0.78 and 0.80, respectively, which are reliable because value is above 0.7, as recommended by Kline [18].

A productivity data sheet was used to assess the rate of work done and the outcome that was achieved within a stated time, during the three different time of the day in the course of farming operation. The productivity measured was with respect to the count made based on the number of ridges tilled or hoed per hectare by a farmer. The farmers were observed and their corresponding productivity output was recorded after every 3 hours of tilling operation. The farming operation normally starts by approximately 6 am which last up to 3 pm.

Anthropometric data were measured using a weighing scale and tape height instrument, based on the gold standard method [19]. The height of the respondents was measured in meters with the respondents standing upright barefoot, while the weight was measured in kilogram with the respondents standing barefoot on a digital meter. A 3M QuesTemp° 36 wet bulb globe temperature (WBGT) monitor was used in measuring the heat index, ambient temperature, relative humidity, air movement, and radiant heat, which is calculated and displayed as a single WBGT index value after considering the above four environmental parameters in order to assess human stress. The instrument was placed in the work area 3.5 feet off the ground using a tripod stand and was allowed for ten minutes for sensors to stabilize before pressing the Run/Stop key for data logging each day. The device logged data from the four sensors after every 5 minutes all day and then automatically calculated the WBGT (outdoor) and stored the results. The data were downloaded to a computer and recorded in an electronic form at the end of the data collection. The device was calibrated daily by plugging the verification module device into the sensor at the top of the unit; the displayed readings were then verified to match those printed on the module within ±0.5°C.

### 2.3. Data Analysis

Descriptive statistics was used in analysing the following data: sociodemographic characteristics, anthropometric data of the respondents, self-reported heat stress-related illnesses or symptoms, and environmental heat stress index. The differences in productivity between the three different times of the day during the farming operation were determined using one-way ANOVA and Tukeyʼs post hoc analysis. Lastly, multiple linear regression was used to determine the influence of temperature (WBGT), body mass index (BMI), age, and gender on the productivity of the farmers.

### 2.4. Ethical Statement

Before the commencement of the study, an ethical clearance with reference code of UPM/INCPI/RMC/1.4.18.2 was obtained from the Research Ethics Committee, JKEUPM (Jawatankuasa Etika Universiti Untuk Penyelidikan Melibatkan Manusia), University Putra Malaysia, and was approved. In addition, prior to the administration of the questionnaires, all the participants have been issued a consent form informing them on the nature of the study and must agree before partaking in the study. The data obtained in the study were strictly kept confidential.

## 3. Results

### 3.1. Sociodemographic Characteristics and Anthropometric Data

Table 1 presents the distribution of the respondents by their sociodemographic characteristics and anthropometric data. The results showed that the majority of the respondents were males, 251 (63.46%) of the sample population. The age of the respondents ranged from 18 to 55 years, with a mean of 30.6 (7.83). Most of the respondents had secondary school education (62.9%). However, 10.6% of them had no formal education. The respondents' years of experience in farming were between 3 and 33 years with a mean (SD) of 14.39 (6.77) years and had cultivated a hectare of land with a mean of 2.70 (0.95). Respondents' body mass index (BMI) was within the range of 17.30–35.55 kg/m^2^, with the mean (SD) of 25.83 (3.73) kg·m^−2^.

### 3.2. Environmental Parameters Measurement (WBGT)

The results of the heat index of the farms by the wet bulb globe temperature device for the environmental parameters, air temperature, radiant heat humidity, and wind movement, showed that the minimum and maximum temperature ranges from 23–36°C between 6 am and 3 pm, as shown in Table 2.

### 3.3. Self-Reported Heat Stress-Related Illnesses (HRI) or Symptoms

Table 3 presents the results of respondents' self-reported heat stress-related symptoms. The majority of the respondents experienced the early symptoms of heat stress such as heavy sweating 369 (93.20%), heat rash (34.30%), tiredness (76.80%), dizziness (76.30%), headache (71.20%), elevated body temperature, and rapid pulse. However, late symptoms such as nausea/vomiting, muscle cramp, fainting, hot dry skin, and unconsciousness were rarely or never experienced at all.

### 3.4. Comparison of Productivity between the Hours of 6–9 am, 9 am–12 pm, and 12–3 pm

A one-way ANOVA was conducted to determine the mean difference between three different times of the working hours of the day on the productivity of farmers. There was a statistically significant difference on the farmersʼ productivity during the three times of the working hours (*p* < 0.001). Productivity was significantly higher between the hours of 6–9 am (*p* < 0.001) and 12–3 pm (*p* < 0.001), compared to the hours of 9 am–12 pm (*p* < 0.001), as shown in Table 4.

### 3.5. The Predictors of Farmers' Productivity: Temperature (WBGT), Gender, Age, and Body Mass Index (BMI)

Table 5 presents the results of the predictors of farmers productivity: temperature, gender, age, and BMI. A significant regression was calculated for all the variables tested, where temperature (*p* < 0.001), gender (*p* < 0.001), age (*p*=0.003), and BMI (*p*=0.008) have a significant relationship with productivity.

## 4. Discussion

The majority of the respondents were males (63.4%), cultivating hectares of land with the mean of 2.7. (0.95). The mean BMI of the respondents was 25.83 (3.73) showing slightly overweight. The highest prevalence of HRI or symptoms reported among workers experienced every day and on alternated days were heavy sweating, tiredness, dizziness, and headache. These might be attributed to exposure to the high amount of heat through working under direct sunlight for almost 8 hours per day. These findings were in agreement with [20], where he found that heavy sweating and headache and weakness/fatigue were the most commonly reported among farmers in Oregon. A study showed a significant association between the high temperature in Thailand and the rate of occupational injuries, especially among agricultural workers [21]. The findings were still in conformity with that of [22] where heat fatigue, dizziness, muscle cramp, and heat rash were found to be the highest prevalence of HRI. The lowest case reported was heat syncope and heat stroke. The study [23] also showed that the highest prevalence of HRI and symptoms were heat exhaustion, heat cramp, and heat rashes. The low prevalence's were heat syncope and heat stroke. Another study [24] reported 40% of participants with heat stress-related symptoms, 22% reported muscle cramp, and only 1% had experienced fainting.

The maximum and mean WBGT heat stress index recorded between the hours of 9 am–12 pm and 12–3 pm exceeded the threshold limit for a moderate physical activity, which is 32.28°C for light work, 31.18°C for moderate work, and 30.08°C for heavy work [25]. Therefore, workers were subjected to heat stress for about one-third hours of their daily work. Thus, it was concluded that heat stress index at this level in agricultural activities causes heat strain and reduced work productivity [26]. A previous study showed work capacity rapidly reduces as the WBGT exceeds 26–30°C [27]. The findings of this study showed a statistically significant difference in productivity based on the number of ridges cultivated between the three different times of the working hours of the day, whereby the productivity is high in the early morning hours compared to the afternoon hours. This difference in productivity could be related to the increase in temperature between these hours. The WBGT between the hours of 6 am to 9 am ranged from 23 to 27°C, which is within the threshold level, recording the highest productivity outcome of 2–17 ridges. Compared to the hours of 9 am–12 pm when the WBGT is 28–35°C, which is above the threshold level, the records showed lesser productivity of 1–9 ridges. Furthermore, the least productivity of only 1 to 4 ridges was recorded between 12 and 3 pm when the WBGT increased from 29 to 36°C. Thus, the productivity decreases by 45% (3 ridges) and 74% (5 ridges) from 9 am to 12 pm and 12 to 3 pm, respectively, as compared to the hours of 6–9 am. The productivity also decreases by 54% (2.20 ridges) between 12 and 3 pm as compared to that of 9 am to 12 pm.

Previous studies estimated that above 27°C any one-degree change in temperature is associated with 4–8% decline in productivity [28]. Similarly, work capacity rapidly reduces whenever temperature exceeds 26–300 [27]. Likewise, 7% increase in work productivity is recorded at temperatures above 20–24°C [29]. A study [30] also showed that 2% loss in productivity is recorded for every degree rise in temperature. Finally, productivity is affected after about an hour of moderate physical work in temperatures above 32°C [31]. A significant multiple linear regression was found for all the variables (WBGT, BMI, age, and gender), with an *R*^2^ of 0.444 (44.4%). This indicated that the 4-factor model explained 44.4% of the variance in productivity. The finding showed that for every additional one unit increase in productivity, −1.040 decrease in temperature (WBGT) is expected, indicating a negative linear relationship, and the same was with BMI where for every additional increase in productivity, −0.064 decrease was expected in BMI. However, age and gender, unlike WBGT and BMI, showed a positive linear relationship for every unit increase in productivity, 0.023 increase was expected in age and for every male worker, 0.873 increase is expected in his productivity as compared to females.

The reduction in productivity as a result of increase in WBGT might be due to the fact that heat causes tiredness/weakness coupled with other heat-related illnesses or symptoms that can even make a person under such condition to seek for a more comfortable thermal environment, thereby slowing down and/or stopping work task completely in order to reduce internal heat production and heat exhaustion [32]. Related studies found a similar result, where work productivity decreased gradually per hour with a corresponding increase in temperature, and about 30% of work output is lost hourly, for every 5°C increase in WBGT [26]. A study [33] showed that for every 1°C increase in WBGT, productivity decreased by 0.57%. Also, heat stress reduces productivity by 0.33% when the WBGT increased by 1°C [34]. Similarly, about 20% of work capacity is lost at a WBGT of 28°C, 30°C, and 32°C for heavy, moderate, and light work, respectively [35]. Another study showed a statistically significant relationship, which recorded increase in WBGT with a corresponding decrease in productivity [36]. The reduction in productivity was seen because the natural response of a working person to heat is to reduce physical activity. This is in order to reduce the body's internal heat production and prevent ill health. The result of this action will subsequently reduce hourly work capacity and lower productivity [37].

The explanation for the decrease in productivity as BMI increased might be attributed to the increase in metabolic rate. This finding coincides with the study of Nigatu et al. [38], where their findings showed that overweight and obesity might reduce performance and limit the ability to accomplish a task successfully. Likewise, the findings coincided to a lesser magnitude where BMI was found to have a weak negative relationship with the productivity of workers [39]. Another study showed that heat exhaustion is more prevalent among higher BMI group compared to the moderate group [40].

The majority of the workers fall within the youthful age, thereby making it logical that as age increases with the sample population, productivity also increases because youth exercise more physical work compared to teenagers and the elderly. Previous findings showed agreement where physical activity decreases at the early stage of the adolescent [41]. A study [42] reported that older workers over the age of 45 years had the higher physiological cost of work and lower ability to work in a hot environment than younger workers. Another study showed that productivity decreased by 0.72% for every one year increase in age [33]. The difference in the physiological makeup of both genders (male and female) determines their endurance and level of physical exertion, thus, determining their output. Similar studies showed that, in most cases, male farmers achieved higher productivity compared to female farmers [43, 44].

## 5. Conclusion

The findings showed that HRI and symptoms frequently experienced almost every day by the majority of the respondents were heavy sweating, tiredness, dizziness, and headache. Findings showed that farmers were under heat stress mostly during 9 am–12 pm in the morning and 12–3 pm of the day, as the heat stress index of the environment exceeded the threshold level set by ACGIH for moderate physical activity. A lower WBGT, male, older, and lower BMI of respondents significantly contributed to higher productivity of the maize farming. The findings showed that high temperature had reduced the productivity of these farmers. This study recommends that farmers should continue to adhere to the use of PPE, such as wear light cotton breathable clothing, which can absorb their perspiration and sunhat for protection from direct sunlight. They should constantly and frequently drink water even without feeling thirsty, so as to stay hydrated. They should work mostly during the early morning hours of the day (6 am–12 pm) in order to achieve high productivity outcome. The study recommends future research to focus on other study designs such as prospective study design and extend the research to other farmers not just maize. This study only examined one activity of maize farming operation (weeding), thus future research should focus on other operations from land clearance to harvesting.

## Figures and Tables

**Table 1:** Distribution of respondents according to sociodemographic characteristics and anthropometric data (*n*=396).

Sociodemographic characteristics/anthropometric data	Mean (SD)	*n* (%)
Gender		
Male		251 (63.40)
Female		145 (36.60)
Age (years)	30.60 (7.83)	
Level of education		
Nonformal education		42 (10.60)
Primary school		37 (9.30)
Secondary school		249 (62.90)
Tertiary level		68 (17.20)
Average monthly income (*N*)	21,070.70 (5177.85)	
Marital status		
Married		203 (51.30)
Single		193 (48.70)
Number of children	1.74 (2.26)	
Years of experience	14.39 (6.77)	
Hectares cultivated	2.70 (0.95)	
Height (m)	1.60 (0.10)	
Weight (kg)	66.92 (8.90)	
BMI (kg/m^2^)	25.83 (3.73)	

**Table 2:** Environmental parameters measurement (WBGT).

Time range	Temperature (°C)	
	Range	Mean (SD)
6 am–9 am	23–27	25.16 (1.33)
9 am–12 pm	28–35	**31.56 (2.19)**
12 pm–3 pm	29–36	**34.08 (1.54)**

**Table 3:** Respondents' self-reported heat stress-related symptoms.

S/N	Items	1	2	3	4	5
1	Heavy sweating as a result of heat stress	0 (0)	0 (0)	7 (1.80)	20 (5.10)	369 (93.20)
2	Heat rash/pricking sensation	154 (38.90)	91 (23.00)	15 (3.80)	18 (4.50)	118 (29.80)
3	Tiredness/weakness as a result of heat stress	3 (0.80)	22 (5.60)	67 (16.90)	112 (28.30)	192 (48.50)
4	Dizziness as a result of heat stress	35 (8.80)	23 (5.80)	36 (9.10)	167 (42.20)	135 (34.10)
5	A headache as a result of heat stress	6 (1.50)	85 (21.50)	23 (5.80)	122 (30.80)	160 (40.40)
6	Rapid pulse as a result of heat stress	36 (9.10)	48 (12.10)	156 (39.40)	56 (14.10)	100 (25.30)
7	Nausea/vomiting as a result of heat stress	167 (42.20)	54 (13.60)	23 (5.80)	18 (4.50)	134 (33.80)
8	Elevated body temperature as a result of heat stress	10 (2.50)	26 (6.60)	221 (55.80)	61 (15.40)	78 (19.70)
9	Muscle cramp as a result of heat stress	95 (24.00)	153 (38.6)	28 (7.10)	81 (20.50)	39 (9.80)
10	Fainting as a result of heat stress	289 (73.00)	25 (6.30)	37 (9.30)	1 (0.30)	44 (11.10)
11	Hot dry skin as result of heat stress	202 (51.00)	118 (29.80)	49 (12.40)	27 (6.80)	0 (0)
12	Difficulty in breathing as a result of heat stress	288 (72.70)	59 (14.90)	47 (11.90)	2 (0.50)	0 (0)
13	Unconsciousness as a result of heat stress	386 (97.50)	9 (2.30)	1 (0.30)	0 (0)	0 (0)

Note: 1 = never, 2 = rarely (once a month), 3 = fairly often (once a week), 4 = often (alternate days), and 5 = very often (everyday).

**Table 4:** Comparison in productivity between the hours of 6–9 am, 9 am–12 pm, and 12–3 pm.

	Time of different work phase	Productivity			
		Mean (SD)	(df)	*F*	*p*
1	6–9 am	7.52 (3.56)	(2, 1185)	557.59	**<0.001**
2	9 am–12 pm	4.11 (1.88)			
3	12–3 pm	1.89 (0.93)			

^*∗*^One-way ANOVA. Post hoc analysis: a Tukey post hoc analysis indicated that the productivity was statistically significant higher between the hours of 6–9 am 7.52 (3.56) and the hours of 12–3 pm 1.89 (0.93), (*p* < 0.001), compared to the hours of 9 am–12 pm 4.11 (1.88), (*p* < 0.001).

**Table 5:** The predictors of farmers' productivity.

	Adjusted coefficients				Crude coefficients			
	B	S.E	Beta		B	S.E	Beta	
WBGT	−1.040	0.066	−0.600	**<0.001**	−1.058	0.069	−0.610	**<0.001**
BMI	−0.064	0.024	−0.119	**0.008**	−0.120	0.027	−0.222	**<0.001**
Age	0.023	0.011	0.089	**0.033**	0.000	0.013	0.001	0.983
Gender	0.873	0.169	0.208	**<0.001**	1.032	0.205	0.246	**<0.001**

^*∗*^Significant at *p*=0.05 level. *R*^2^ = 0.444: the four factor model (WBGT, gender, age, and BMI) explained 44.4% of the variance in productivity for multiple regression model.

## Data Availability

This study only used previous findings from published work and is referenced within the text.
